# Therapeutic Applications of Cysteamine and Cystamine in Neurodegenerative and Neuropsychiatric Diseases

**DOI:** 10.3389/fneur.2019.01315

**Published:** 2019-12-12

**Authors:** Bindu D. Paul, Solomon H. Snyder

**Affiliations:** ^1^The Solomon H. Snyder Department of Neuroscience, Johns Hopkins University School of Medicine, Baltimore, MD, United States; ^2^Department of Psychiatry and Behavioral Sciences, Johns Hopkins University School of Medicine, Baltimore, MD, United States; ^3^Department of Pharmacology and Molecular Sciences, Johns Hopkins University School of Medicine, Baltimore, MD, United States

**Keywords:** BDNF, brain, cystamine, cysteamine, cysteine, neurodegeneration, neuropsychiatric disorder, redox

## Abstract

Current medications for neurodegenerative and neuropsychiatric diseases such as Alzheimer's disease (AD), Huntington's disease (HD), Parkinson's disease (PD), and Schizophrenia mainly target disease symptoms. Thus, there is an urgent need to develop novel therapeutics that can delay, halt or reverse disease progression. AD, HD, PD, and schizophrenia are characterized by elevated oxidative and nitrosative stress, which play a central role in pathogenesis. Clinical trials utilizing antioxidants to counter disease progression have largely been unsuccessful. Most antioxidants are relatively non-specific and do not adequately target neuroprotective pathways. Accordingly, a search for agents that restore redox balance as well as halt or reverse neuronal loss is underway. The small molecules, cysteamine, the decarboxylated derivative of the amino acid cysteine, and cystamine, the oxidized form of cysteamine, respectively, mitigate oxidative stress and inflammation and upregulate neuroprotective pathways involving brain-derived neurotrophic factor (BDNF) and Nuclear factor erythroid 2-related factor 2 (Nrf2) signaling. Cysteamine can traverse the blood brain barrier, a desirable characteristic of drugs targeting neurodegeneration. This review addresses recent developments in the use of these aminothiols to counter neurodegeneration and neuropsychiatric deficits.

## Introduction

Cysteamine, also known as 2-mercaptoethylamine or aminoethanethiol, is the decarboxylated derivative of the amino acid cysteine. It exerts radioprotective effects and is more effective than cysteine alone, although a combination of cysteamine and cysteine display synergistic effects (1, 2). Although cysteamine reduced mortality in irradiated *Drosophila* and mice, mutagenic effects of radiation were not prevented (3, 4). Cysteamine has been utilized for the treatment of cystinosis, a lysosomal disorder, and, more recently, has been evaluated for the treatment of neurodegenerative disorders. This review will summarize the current understanding of cysteamine and cystamine, its oxidized derivative.

**Figure 1:**
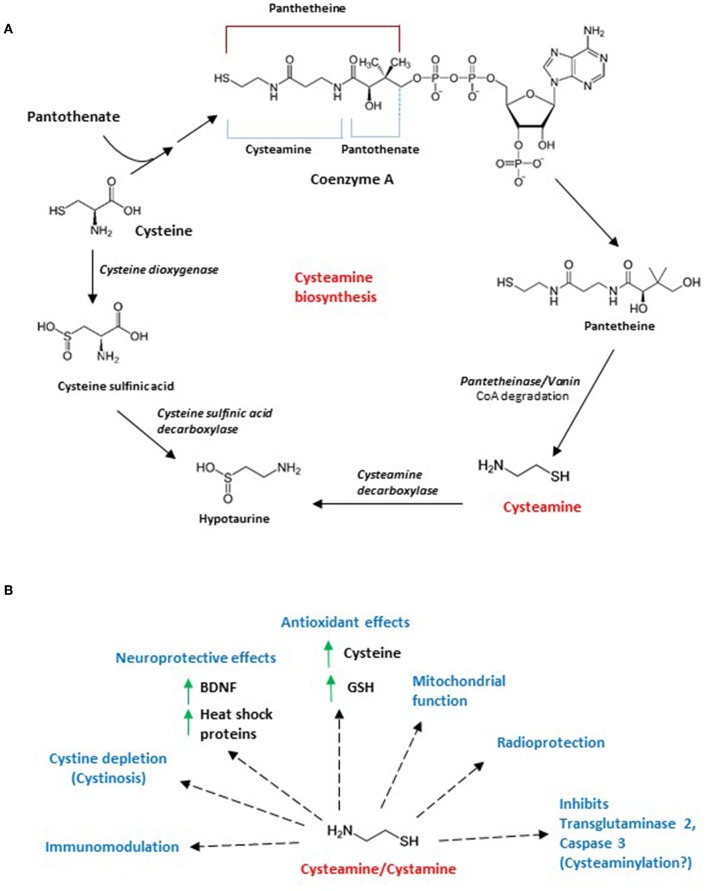
**(A)** Biosynthesis of cysteamine and intersection with cysteine catabolism. Cysteamine is generated in mammals by the degradation of coenzyme A, which is required for the metabolism of fatty acids, carbohydrates, amino acids and ketone bodies. When coenzyme A is cleaved (cleavage at the dotted line), pantetheine is generated, which is acted on by pantetheinase or vanin to form cysteamine. Cysteamine is converted to hypotaurine by cysteamine decarboxylase. Cysteine, a component of coenzyme A, is acted on by cysteine dioxygenase to form cysteine sulfonate which is decarboxylated by cysteine sulfonate decarboxylase to form hypotaurine. Hypotaurine generated is further metabolized to taurine by hypotaurine decarboxylase. **(B)** Effects of cysteamine/cystamine. Both cysteamine and its oxidized form cystamine have protective effects in cells and tissues. Originally identified as radioprotective molecules, subsequently these aminothiols have been reported to mitigate cystinosis, a condition characterized by accumulation of cystine crystals in the body. Cystamine and cysteamine have a variety of other effects which include antioxidant effects (by increasing cysteine and glutathione levels), inhibition of transglutaminase 2 and caspase 3 (possibly by modifying reactive cysteine residues or cysteaminylation), modulation of mitochondrial function, immunomodulation. These molecules have also been reported to increase levels of brain derived neurotrophic factor (BDNF) and heat shock proteins, which affords neuroprotective benefits.

In cells, the amino thiol is generated by the degradation of coenzyme A, which in turn, is generated from pantothenate (vitamin B5) and cysteine (Figure 1A) (5). Coenzyme A degradation yields pantetheine, which is hydrolyzed by pantetheinase or vanin, generating cysteamine and pantothenic acid. Cysteamine is then oxidized to hypotaurine by cysteamine dioxygenase (6). Hypotaurine can be converted into taurine by hypotaurine dehydrogenase. Taurine is eliminated in the form of bile salts such as taurocholate, either via the urine or feces (7). Levels of cysteamine has been reported to be in the low micromolar range in tissues such as the liver, kidney and brain, which were measured after treating lysates with DTT to liberate free cysteamine (6), indicating association with proteins via disulfide bonding. Similarly, another study measured cysteamine after reducing perchloric acid treated kidney and liver lysates with mercaptopropionic acid (8). The presence of disulfide-bonded cysteamine with proteins was subsequently shown by Duffel and associates (9), which could account for the effects of cysteamine and cystamine on the activity of several proteins.

The metabolism of cysteamine, cystamine and cysteine are linked in cells. Both cysteamine and cystamine increase cysteine levels intracellularly in a temporal and dose-dependent manner (10). As cysteine is a component of glutathione and a potent antioxidant itself, treatment of cells with these aminothiols can mitigate oxidative stress. Treatment of SN56 cholinergic cells causes an increase in cysteine levels in 30 min. Cystamine is first converted to cysteamine in the reducing atmosphere of cells, and treating cells with cystamine elicits an increase of cysteine in 3 h. N-acetylcysteine (NAC), 2-mercaptoethanesulfonic acid (MESNA) and mercaptopropionylglycine (MPG), on the other hand, elevate cysteine levels to a lesser extent (2-fold as compared to 6-fold in the case of cysteamine). The study also revealed the importance of these thiols in sequestering reactive aldehyde species in cells and bolstering the antioxidant capacity of cells. Thus, cystamine and cysteamine also act as antioxidants themselves. Consistent with these observations, cysteamine affords protection against acetaminophen- mediated liver damage, where the highly toxic unsaturated aldehyde acrolein, is produced (11, 12). Cysteamine has also been proposed to replace homocysteine as the substrate for cystathionine β-synthase (CBS) in a reaction with serine to generate thialysine or (S-(2-aminoethyl)-L-cysteine) (13). Consistent with these studies, thialysine levels increase in the brain after feeding cysteamine to rats (14).

## Protective Effects of Cysteamine and Cystamine

### Therapeutic Applications of Cysteamine and Cystamine in Peripheral Tissues

Both cysteamine and cystamine, have been used for the treatment of several conditions (Figure 1B). These compounds possess radioprotective properties and were initially used to treat radiation sickness that arises in cancer patients after radiotherapy, but subsequently discontinued after unsuccessful clinical trials (1, 15). One of the earliest uses of cysteamine in medicine, which is FDA-approved, is the treatment of cystinosis, an inherited autosomal recessive disorder in which the body accumulates cystine due a defect in the lysosomal cysteine transporter, cystinosin (16, 17). Cystine crystals build up in many tissues and damage organs such as the kidney and the eye. One of the initial manifestations of juvenile cystinosis is renal Fanconi syndrome which manifests as dysfunction of the renal proximal tubule leading to polyuria, phosphaturia, glycosuria, proteinuria, acidosis, growth retardation, and rickets (18). Cysteamine participates in disulfide exchange reactions to form cysteine and mixed disulfides of cysteine and cysteamine, which can then exit the lysosome.

Cysteamine also has anti-malarial effects preventing the replication of the parasite, *Plasmodium falciparum in vivo* and also potentiates the action of the anti-malarial artemisin (19, 20). Cysteamine has also been reported to have anti-HIV-1 effects (21, 22). Cysteamine elicits both beneficial and harmful effects, some of which included ulcer formation and anti-angiogenic effects (23). Cystamine, the oxidized form of cysteamine, inhibits erythrocyte sickling in sickle cell anemia (24). Incubating sickle cells with cystamine leads to the formation of an S-ethylamine derivative and a decrease in sickling under hypoxic conditions. Several other beneficial effects of the two cysteine derivatives are summarized in Table 1.

**Table 1:** Neuroprotective actions of cysteamine/cystamine.

**Cytoprotective effects of Cysteamine/Cystamine**	**System**	**References**
Protection against glutamate-induced toxicity	Primary glial cells	(25)
Scavenges acrolein, a toxic metabolite generated during lipid peroxidation. Drug detoxification and polyamine oxidation	Cysteamine: Acetaminophen-induced hepatic injury in mice	(26)
Reduces oxidative stress and antioxidant balance in regulatory T cells	Cystamine: systemic lupus erythematosus (SLE)-prone mice	(27)
Improved membrane functionality, reduced lipid peroxidation and improved viability of sperm	Cysteamine: Cyropreserved Ram semen	(28)
Intraperitoneal injection of cystamine mediates neuroprotection by enhancing neuronal progenitor cell proliferation and proliferation through the BDNF pathway	Cystamine: mouse model of stroke	(29)
Dopaminergic neurodegeneration induced by MPTP is prevented by cysteamine and cystamine	MPTP model of neurodegeneration	(30, 31)
Neuroprotection from 3-nitropropionic acid (3NP) toxicity by cystamine	Stimulates NF-E2 related factor 2 (Nrf2) signaling in cell culture and the 3-NP model of neurodegeneration in mice	(32)
Administration of cystamine confers protection against haloperidol-induced toxicity and ischemic brain injury	Mouse model	(33)
Aggregation of amyloid β_1−42_ (Aβ) in astrocyte cultures reduced by cystamine	Cultured astrocystes	(34)
Cystamine elevated L-cysteine levels in HD	R6/2 mouse model of HD and PC12 model of polyglutamine aggregation	(35)
Transglutaminase-induced aggregation of alpha-synuclein decreased by cystamine	*in vitro* and in COS-7 cells	(36)
Cystamine significantly extended survival, improved body weight and motor performance, and delayed the neuropathological sequela	R6/2 mouse model of HD	(37)
Cystamine increased viability of striatal progenitor cells harboring mutant huntingtin and prevented ROS formation in HD cells subjected to H2O2 and STS	STHdh^Q7/Q7^ and STHdh^Q111/Q111^ striatal progenitor cell lines	(38)
Cysteamine and cystamine prevented the 3-NP-mediated decrease in cellular and mitochondrial GSH levels as well as mitochondrial depolarization	STHdh^Q7/Q7^ and STHdh^Q111/Q111^ striatal progenitor cell lines	(39)
Cystamine extended survival, reduced associated tremor and abnormal movements and ameliorated weight loss. Increased the transcription of the chaperone HDJ1/Hsp40	R6/2 mouse model of HD	(40)
Cystamine significantly delayed the progression of ALS symptoms and reduced SOD1 oligomers and microglial activation	G93A mouse model of ALS, cell culture models	(41)
Cystamine prevents toxicity induced by aggregation of polyadenylate-binding protein nuclear 1	Mouse model of Oculopharyngeal muscular dystrophy (OPMD)	(42)
Cystamine modulates blood pressure and reduces hypertension	Spontaneously hypertensive rats	(43)
Cysteamine alleviates fibrosis and symptoms associated with chronic kidney disease (CKD)	Mouse models of CKD	(44)
Cysteamine suppresses cataract formation induced by selenite	Rats	(45)
Cystamine rescued behavioral deficits induced by 2,5-hexanedione by increasing BDNF and hsp70 expression	Rats	(46)

### Therapeutic Applications of Cysteamine and Cystamine in Brain Diseases

Cysteamine and cystamine appear to be promising in the treatment of certain mouse models of neurodegenerative diseases, such as Parkinson's disease (PD) and Huntington's disease (HD) (47). Cysteamine can cross the blood-brain barrier, which makes it an attractive candidate for therapeutic applications (48).

#### Huntington's Disease

Huntington's disease is a neurodegenerative disorder caused by expansion of polyglutamine repeats in the protein huntingtin, Htt, which causes it to aggregate and cause widespread damage in almost all tissues expressing it (49). Initial studies on cystamine and its therapeutic effects on disease progression in HD focused on its inhibitory effects on the enzyme transglutaminase (37, 40). Transglutaminases catalyze the formation of ε-N-(γ-glutamyl)-lysyl crosslinks between proteins and were proposed to contribute to neuropathology of HD (50–52). However, later studies revealed that ablation of the transglutaminase gene did not prevent neurodegeneration in HD (53). Cystamine has also been beneficial in a fly model of HD, where photoreceptor degeneration was rescued in adult flies (54). Cystamine treatment in mouse models of HD lead to increased cysteine levels, which was proposed to be neuroprotective (35, 55). Cysteine is a potent antioxidant and dysregulated cysteine metabolism mediates neurodegeneration in HD (56–58). Cysteine is also the precursor of the gaseous signaling molecule, hydrogen sulfide, which participates in a myriad of physiological processes (59–61). Cystamine, in combination with mithramycin, was also shown to be protective in the R6/2 model of HD (62). The beneficial effects of cysteamine led to clinical trials in HD (63). In addition, cystamine can augment levels of brain derived neurotrophic factor, BDNF, in mouse models of HD (64). More recently cysteamine was shown to counteract toxicity mediated by mutant huntingtin *in vitro* in primary neuron and iPSC models of HD although the exact molecular mechanism by which cytoprotection is conferred is still unknown (65).

#### Alzheimer's Disease

Alzheimer's disease (AD) is the most prevalent neurodegenerative disorder and the most common form of dementia (66, 67). The molecular hallmarks of AD include increased load of amyloid plaques and neurofibrillary tangles, which affect multiple cellular processes. Numerous reports describe links between dementia and AD with amyloid deposits or tangles. Postmortem analysis of cognitively normal subjects have revealed increased amyloid plaques, a pathogenic signature of AD, but no dementia (68). Conversely, several diagnosed AD patients have no signs of neuritic plaques (69). Thus, the correlation between amyloid plaques and AD awaits further study (70). Regardless of these inconsistencies, it is clear that the brain has corrective mechanisms that delay cognitive decline and if harnessed, may stall neurodegeneration. The search for small molecules that stimulate neuroprotective signaling cascades may be beneficial. Cystamine and its derivatives are being evaluated as possible therapies for the disease. Chronic cysteamine treatment (daily injections for a period of 4 months) resulted in improvements in habituation and spatial learning deficits in the APP-Psen1 mouse model of AD (71). The APP-Psen1 model harbors the human transgenes for the Swedish mutation of the amyloid precursor protein (APP) and presenilin-1 (PSEN1) containing an L166P mutation, regulated by the Thy-1 promoter (72). AD patients have elevated transglutaminase levels, which colocalize with the amyloid plaques (34). Transglutaminases accelerate amyloid beta aggregation and toxicity. Accordingly, cystamine therapy is being considered for lowering the amyloid plaque burden in AD patients. In particular, Multi-Target Directed Ligands (MTDLs) or single compounds which may simultaneously act on different targets are being explored. Along these lines, a cysamine-tacrine dimer has been developed, which decreased acetylcholinesterase (AChE)-induced beta-amyloid aggregation (73).

#### Parkinson's Disease

Aggregation of alpha-synuclein, leading to the formation of Lewy bodies, is a hallmark of Parkinson's disease (PD), which affects the substantia nigra of the brain causing motor deficits and multiple abnormalities. Existing therapies for PD largely target symptoms and do not mitigate neuronal loss observed. Several lines of evidence suggest the therapeutic potential of the aminothiol in PD (71). Cystamine ameliorated mitochondrial dysfunction and oxidative stress associated with 6-hydroxydopamine and 1-methyl-4-phenyl-1,2,3,6-tetrahydropyridine (MPTP)-induced models of PD (74). In the MPTP-induced neurotoxicity model of PD in mice, independent studies revealed various effects of cystamine such as elevation in the levels of tyrosine hydroxylase and BDNF (30, 75). Similarly, cysteamine, the reduced form of cystamine, also afforded neuroprotection. Similar to AD, elevated transglutaminase activity caused an increase in the formation of cross-linked alpha-synuclein and insoluble aggregates, which could be abrogated by cystamine (36).

#### Amyotrophic Lateral Sclerosis

ALS, also known as Lou Gehrig's disease, is a neurodegenerative disease where selective degeneration of motor neurons in the brain and spinal cord occurs leading to paralysis of skeletal muscles and progressive weakness and atrophy of limbs (76). Difficulties in speech and movement follow and patients are typically wheelchair-bound. Causes of ALS can be either genetic or sporadic (refers to patients without a family history). Among the best studied genetic mutations in familial ALS include mutations in superoxide dismutase 1 (SOD1), which misfolds, aggregates, and elicit toxicity by multiple mechanisms (77, 78). Proposed reasons for SOD1 aggregation include crosslinking mediated by transglutaminase 2 (TG2). Studies with cell culture models of ALS reveal that cystamine prevents aggregation of SOD1 and improved cell survival (79). Furthermore, inhibiting spinal TG2 by cystamine reduces SOD1 oligomers, microglial activation and delayed progression in the G93A SOD1 mouse model of ALS (41). Thus, cystamine treatment may be beneficial in treating ALS.

#### Neurological Complications of Cystinosis

Although cystinosis was not considered to affect brain function, it is now known that cystinosis can result in neurocognitive deficits in adults as well as children. These include impaired visual spatial, visual memory, language problems, academic impairment, seizures, memory impairment, motor incoordination, and neuromuscular dysfunction and is often accompanied by structural abnormalities in the brain (80–82). Early treatment with cysteamine orally prevents several of these neurocognitive deficits. Patients with cystinosis treated at or after age 2 years (late-treatment group) score poorer than the early treatment group (before 2 years) on verbal, performance, and full-scale IQ tests and tests rating visual-spatial skills (83). Similarly, adults with cystinosis who receive consistent chronic treatment with cysteamine fare better on visual learning and memory skills (84).

#### Schizophrenia and Neuropsychiatric Diseases

Schizophrenia is a psychiatric disease, with complex genetic and neurological contributions of unclear origins, manifesting as a combination of symptoms which includes hallucinations, delusions, motivational and cognitive deficits (85). Although treatments for schizophrenia target psychotic symptoms, most existing drugs do not relieve social and cognitive deficits. The neurochemical changes in schizophrenia typically occur well before formal diagnosis, and, thus, preventive therapies could be beneficial. Schizophrenic patients have lower levels of BDNF so that schizophrenic patients might benefit from use of cysteamine due to its BDNF-enhancing properties and effects on the dopaminergic system (86, 87). In an amphetamine-induced psychosis model of schizophrenia, cysteamine prevents increased locomotor activity by decreasing dopamine release (88). Cysteamine counteracts the BDNF-lowering effects of haloperidol (89). The anti-depressant effect of cysteamine may also benefit other mental conditions (90). These studies are consistent with an earlier study which demonstrated that cysteamine blocked amphetamine-induced deficits in sensorimotor gating in male Sprague-Dawley rats (91). Similarly, cysteamine treatment increases BDNF levels in the frontal cortex and hippocampus and improved spatial memory in heterozygous reeler mice, which exhibit behavioral and neurochemical abnormalities similar to those in schizophrenia (92).

Similarly, cystamine and cysteamine may be beneficial in other conditions involving low neurotrophin levels, such as autism spectrum disorders (ASD). Analysis of postmortem human brain samples revealed increases in TG2 mRNA and protein levels in the middle frontal gyrus of subjects with autism spectrum disorder. Thus, cysteamine may alleviate symptoms of ASD by inhibiting TG2 and increasing BDNF levels (93). The same study demonstrated that ER stress induced TG2 expression and deficits in social behavior. Systemic administration of cysteamine attenuated these behavioral abnormalities. In mice lacking methyl-CpG binding protein 2 (MeCP2), a model of Rett syndrome, associated with decreased BDNF levels and obsessive compulsive phenotypes, cysteamine treatment improved lifespan, and improved motor function (94, 95). In a similar vein, cysteamine counteracted anxiety, and depression-like behaviors in a mouse model of anxiety/depression induced by chronic glucocorticoid exposure (96).

## Potential Side-Effects of Cysteamine and Cystamine

Although cysteamine and cystamine have several desirable effects in cells and tissues, some studies have reported side-effects. For instance, in the treatment of HD patients using cysteamine (Cystagon) in the CYTE-I-HD clinical trials, rashes, nausea, and motor impairment along with bad breath were observed in a few patients (63). In phase II trials, asthenia or fatigue was more commonly observed (97). Despite these side-effects, cysteamine appeared to be well tolerated by almost all of the patients.

## Concluding Remarks

Some therapies using antioxidants have not yielded satisfactory outcomes in clinical trials (98–101). Several reasons have been attributed to the failure of such trials. Certain antioxidants inhibit fundamental cellular processes such as autophagy, which is crucial to eliminate misfolded proteins and damaged organelles (102). Most antioxidants utilized only target specific free radicals and thus may counteract only selected types of free radicals. Most clinical trials were initiated relatively late in disease progression, when most of the oxidative damage has already accrued. Doses of antioxidants utilized have also not been adequately tested. Durations of several of these trials have also been short, and longer term uses of redox active molecules have not been studied in detail. Thus, development of antioxidant molecules that have multiple targets, while not inhibiting basic cellular processes such as autophagy, is crucial. Cysteamine normalizes the proteostasis machinery by restoring BECN1/Beclin 1-dependent autophagy in cystic fibrosis in mouse models of the disease and also in patients (103). Cysteamine dendrimers have been found to ameliorate autophagy deficits in cystic fibrosis (104). It is evident that signaling pathways modulated by cystamine and cysteamine are diverse (Figure 1B), and knowledge of these cascades will yield information that can be harnessed to tailor treatments for diverse diseases. The tissue-specific effects and optimal concentrations of the thiol redox couple that would be beneficial for specific diseases has still not been elucidated. Although these aminothiols possess beneficial disease-modifying effects in several conditions, it is still unclear whether these molecules or their metabolites mediate the cytoprotection observed in neurodegenerative diseases. However, increase in cysteine levels can promote neuroprotection, and some of the beneficial effects can be attributed to increases in cysteine to mitigate oxidative stress as has been observed in HD (56). Similarly, systematic studies measuring the concentration and metabolism of cysteamine and cystamine in pathological conditions have not been conducted and are areas of future investigation. Epigenetic effects of cystamine and cysteamine and cysteaminylation, the posttranslational modification mediated by cystamine and cysteamine await detailed investigation. The use of cystamine and cysteamine is another example of a repurposed drug, which has cytoprotective effects in the brain. Combination therapy of these aminothiols with other approved drugs offer attractive options to arrive at safe and effective drugs for these complex diseases.

## Author Contributions

BP conceptualized the review. BP and SS wrote the review.

### Conflict of Interest

The authors declare that the research was conducted in the absence of any commercial or financial relationships that could be construed as a potential conflict of interest.

## Abbreviations

AChE: acetylcholinesterase
AD: Alzheimer's disease
APP: amyloid precursor protein
ALS: Amyotrophic lateral sclerosis
ASD: Autism spectrum disorders
BDNF: brain-derived neurotrophic factor
BECN1: beclin 1
CBS: cystathionine β-synthase
HD: Huntington's disease
MPTP: 1-methyl-4-phenyl-1,2,3,6-tetrahydropyridine
MESNA: 2-mercaptoethanesulfonic acid
MPG: mercaptopropionylglycine
MeCp2: methyl-CpG binding protein 2
MTDL: multi-target-directed ligand
PD: Parkinson's disease
Nrf2: Nuclear factor erythroid 2-related factor 2
PSEN1: presenilin 1
SOD1: superoxide dismutase 1
TG2: transglutaminase 2.
